# High intensity interval training promotes total and visceral fat mass loss in obese Zucker rats without modulating gut microbiota

**DOI:** 10.1371/journal.pone.0214660

**Published:** 2019-04-09

**Authors:** Florie Maillard, Emilie Vazeille, Pierre Sauvanet, Pascal Sirvent, Lydie Combaret, Antoine Sourdrille, Vivien Chavanelle, Richard Bonnet, Yolanda Fernandez Otero, Geoffrey Delcros, Nicolas Barnich, Nathalie Boisseau

**Affiliations:** 1 Université Clermont Auvergne, Laboratoire des Adaptations Métaboliques à l’Exercice en conditions Physiologiques et Pathologiques (AME2P), Clermont-Ferrand, France; 2 Université Clermont Auvergne/Inserm U1071; USC-INRA 2018, Microbes, Intestin, Inflammation et Susceptibilité de l'Hôte (M2iSH), Clermont-Ferrand, France; 3 Université Clermont Auvergne, Inserm, 3iHP, CHU Clermont-Ferrand, Service d’Hépato-Gastro Entérologie, Clermont-Ferrand, France; 4 Université Clermont Auvergne, CHU Clermont-Ferrand, Service de chirurgie digestive, Clermont-Ferrand, France; 5 Université Clermont Auvergne, INRA, UNH, Unité de Nutrition Humaine, CRNH Auvergne, Clermont-Ferrand, France; 6 Department of Bacteriology, CHU Clermont-Ferrand, Clermont-Ferrand, France; Universidade do Estado do Rio de Janeiro, BRAZIL

## Abstract

**Aims:**

Increased visceral adipose tissue and dysbiosis in the overweight and obese promote chronic inflammation. The aim of this study was to compare the effects of moderate-intensity continuous training (MICT) and high-intensity interval training (HIIT) on the gut-adipose tissue cross-talk in obese Zucker rats.

**Methods:**

Obese male Zucker rats (n = 36) were divided in three groups: MICT (12m.min^-1^ for 51min), HIIT (6 sets at 18 m.min^-1^ for 4min followed by 3min at 10m.min^-1^) and controls (CONT; no exercise). The animals ran on a treadmill 5 days/week for 10 weeks. Body composition, glycaemic control, lipid profile, inflammation, lipolysis signalling in subcutaneous and visceral adipose tissue, intestinal permeability (tight junctions and plasma lipopolysaccharide binding protein; LBP), and gut microbiota composition were assessed in the three groups.

**Results:**

After 10 weeks of exercise, total and epididymal fat mass decreased only in the HIIT group. The α/β adrenergic receptor RNA ratio in subcutaneous adipose tissue increased only in the HIIT group. The expression level of phosphorylated hormone-sensitive lipase was not modified by training. Both HIIT and MICT decreased inflammation (plasma myeloperoxidase and keratinocyte-derived chemokine secretion in adipose tissue) and improved glucose metabolism. Zonula occludens-1 and occludin were upregulated in the HIIT group. Plasma LBP was similarly reduced in both training groups. HIIT and MICT did not affect gut microbiota composition.

**Conclusion:**

In obese Zucker rats, HIIT and MICT improved inflammation and glucose metabolism. In contrast, only HIIT decreased total and visceral fat mass. These adaptations were not associated with modifications in gut microbiota composition.

## Introduction

Obesity has dramatically increased worldwide in recent decades and is now a critical health problem. In 2014, 1.9 billion people were overweight and among them 600 million were obese in developed and developing countries [1]. Obesity is characterized by a low-grade inflammation state [2,3]. Abdominal fat mass (FM), especially visceral FM, is associated with metabolic disorders and inflammatory state to a greater extent than total and subcutaneous FM [4]. Visceral FM is characterized by a greater secretory activity (free fatty acids, TNF-α, IL-6, IL-8, *etc*.) that promotes insulin resistance, chronic low-grade inflammation and risks of cardiovascular disease [5,6].

More recently, gut microbiota has been recognized as a major actor in pathological conditions associated with obesity and its related complications [7,8]. This relationship was confirmed by increased adiposity in germ-free mice after faecal microbiota transplant from obese mice (*ob/ob*) [9]. Moreover, a reduced microbial diversity and dysbiosis (*i*.*e*., a microbial imbalance, characterized by an alteration in the Bacteroidetes/Firmicutes ratio) have been observed in overweight or obese individuals [10] and in animal models of obesity [11]. Gut microbiota is an exteriorized organ that can interact directly with other organs. Butyrate, propionate and acetate, the main short-chain fatty acids (SCFAs) produced by microbial fermentation, favourably influence the host metabolism by producing anorexigenic hormones via colonic epithelial cells [12,13], increasing the energy expenditure [14] and reducing inflammation [15]. High-fat diets induce dysregulation of intestinal permeability, promoting an increase in plasma lipopolysaccharide (LPS) (*i*.*e*., a constituent of Gram-negative bacteria), a condition defined as metabolic endotoxemia [16], and triggering inflammation and insulin resistance [17].

Regular physical activity, alone or combined with energy restriction, is an effective way to prevent and/or reduce excess adiposity [18]. Traditionally, Moderate Intensity Continuous Training (MICT) is recommended for the overweight or obese. However, this exercise modality has little effect on weight and FM loss [19,20]. In the last few years, High Intensity Interval Training (HIIT) has grown in popularity as a time-efficient and powerful strategy to reduce total and abdominal/visceral FM [21,22]. Adrenergic receptors (α/β AR ratio) are probably involved in such adaptations, but other, still unknown mechanisms could contribute to FM loss. In this context, modulation of gut microbiota composition by physical activity is an interesting hypothesis. Numerous studies have demonstrated that moderate-intensity training can favourably alter gut microbiota composition in humans [23] and in animal models [24–31]. Most studies with rodents used MICT protocols that involve treadmill running or spontaneous activities on a running wheel. Only two studies investigated the effects of HIIT on gut microbiota composition. Batacan et *al*. found slight differences in gut microbiota phylotype in Wistar rats after MICT and HIIT without any association with FM loss [32]. Similarly, despite changes in gut microbiota composition, Denou et *al*. detected no FM loss after HIIT in C57BL/6 mice [33].

The aim of the present study therefore was to compare the effect of HIIT and MICT programmes on total and visceral FM loss in Zucker rats, a genetic model of obesity. We hypothesized that HIIT is more effective than MICT in decreasing total and visceral FM. We also aimed to determine whether gut microbiota modulation is involved in the reduction of the amount of adipose tissue, and whether the metabolic and inflammatory profiles are improved more effectively by HIIT than by MICT.

## Material and methods

### Ethical approval

The experimental protocol was approved by the “Comité d'éthique en expérimentation animale” of Auvergne (C2EA-02, approval number: 3075–2015120813375547) and was in accordance with the current legislation on animal experimentation (Guide for the care and use of laboratory animals, Eighth edition 2011). All efforts were made to protect animal welfare and to minimize suffering at each step of the protocol. The animals were sacrificed by cervical dislocation following isoflurane anaesthesia.

### Animals

Seventy-five 8-week-old male Zucker rats from Charles River Laboratories were individually housed with a reversed light-dark cycle in a temperature-controlled room (21°C). After 1 week of treadmill acclimatization, rats most proficient at running were selected and randomly assigned to one of the three groups: HIIT (n = 12), MICT (n = 12) or CONT (no exercise)(n = 12).

### Exercise training

Exercise training was performed on a motorized treadmill at 0° inclination 5 days/week (Monday to Friday) for 10 weeks. Both groups started with a warm-up at 10 m.min^-1^ for 5min. In the HIIT group, rats ran 6 sets of 4min at 18m.min^-1^ followed by 3min at 10m.min^-1^. In the MICT group, animals ran at 12m.min^-1^ for 51min. The protocols were originally designed to have the same total running distance for all groups, as proposed by Metz et *al*. 2005 [34], Kapravelou et *al*. 2015 [35] and Haram et *al*. 2009 [36]. CONT rats were placed in the training room during the sessions to expose them to the same environment and for the same time as the HIIT and MICT groups.

### Food intake, weight and body composition

Food (3% fat, 16% protein, 60% carbohydrates, 5% minerals, and 4% fibres; SAFE A04, France) and water were provided *ad libitum*. Food intake was recorded once a week (on Thursday).

Weight was recorded weekly during the 10 weeks of training (W0 to W10). At week 0, 2, 5, 8 and 10, body composition was measured by MRI (Echo Medical Systems, Houston, TX), and epididymal fat pads were weighed *post-mortem*.

### Indirect calorimetry

At week 10 (end of the training), the rats were placed in indirect calorimetric cages (TSE Systems, Bad Homburg, Germany) for 48h (24h of familiarization and 24h of measurements) with *ad libitum* access to food and water. Metabolic measurements (O_2_ and CO_2_ consumption, food intake and spontaneous activity) were recorded. The respiratory exchange ratio (RER) was determined as the ratio of produced CO_2_ (VCO_2_) over consumed O_2_ (VO_2_). Data were analysed over 24h, and in 12h-light and 12h-dark conditions.

### Oral Glucose Tolerance test (OGTT)

OGTTs were performed at the beginning and at the end of the study. After 6h of fasting followed by oral gavage of glucose (4.0 g.kg lean mass^-1^), glycaemia was monitored with a glucometer (Accu-chek Performa, Roche Diagnostics, Basel, Switzerland) and tail blood samples taken at 15, 30, 60, 90 and 120min post-gavage. The area under the curve for glucose (AUC) and the _net_AUC (after subtraction of the baseline glucose concentration) were calculated. The homeostatic model assessment for insulin resistance (HOMA-IR) index was used to assess IR as follows: fasting insulin (mU.L^-1^) × fasting glucose (mmol.L^-1^)/ 22.5 [37]

### Post-mortem blood samples and plasma measurements

At the end of the study, blood was collected and centrifuged at 2000g for 10min for plasma separation. All samples were frozen at -80°C until analysis. Plasma insulin was measured with the Ultrasensitive Insulin ELISA Kit (ALPCO, Salem, NH, USA). Lipid profile was determined by quantifying plasma lipoproteins and lipids with commercial kits following the manufacturers’ instructions: triglycerides (Max Discovery, Austin, USA), LDL (Crystal Chem, Downers Grove, USA), HDL (Crystal Chem, Downers Grove, USA) and total cholesterol (Max Discovery, Austin, USA). LBL plasma level was measured with the LBP ELISA Kit for various species (Hycult Biotech, Netherland) following the manufacturer’s instructions.

### Plasma myeloperoxidase (MPO), and cytokine and free fatty acid (FFA) release from adipocytes

Plasma MPO concentration was measured using a commercial ELISA Kit (R&D Systems).

At the end of the study, adipose tissue was collected and a known amount of each fat pad (subcutaneous and visceral) was placed in Dulbecco's Modified Eagle Medium (DMEM) with antibiotics (50mg.ml^-1^ gentamicin) or in KBEBS-Ringer’s solution (pH = 7.4). After overnight incubation at 37°C, 5% CO_2,_ samples were centrifuged and supernatants frozen at -80°C. Cytokines (KC and IL-6) secreted by tissues were quantified in these supernatants diluted in reagent diluent (1% BSA) using an ELISA Kit (R&D systems). FFA were analysed with a commercial kit (Wako Chemicals, Richmond, USA), according to the manufacturer’s instructions.

### Microbiota composition analysis

Rat colons were transferred in ZR BashingBead Lysis Tubes (0.1 & 0.5 mm, Zymo Research) with lysis buffer (Maxwell RSC Buffy Coat DNA) and homogenized using a Precellys homogeniser (2X 15 seconds followed by 2min rest). Lysis tubes were centrifuged at 14000g at 4°C for 3min, and supernatants were collected in new tubes and centrifuged again to ensure that all beads were removed. The supernatants were then placed in cartridges in Maxwell RSC Instrument (Promega) to extract DNA. DNA concentration was determined by Qubit Fluorometric Quantitation (Invitrogen) and DNA quality was assessed by spectrophotometry (260/280 and 260/230 ratios, Nanodrop). The variable regions V3-V4 of bacterial 16S rRNA genes were amplified from the purified DNA using the following primers: Forward CTTTCCCTACACGACGCTCTTCCGATCTACGGRAGGCAGCAG, and Reverse GGAGTTCAGACGTGTGCTCTTCCGATCTTACCAGGGTATCTAATCCT. All PCR amplifications were performed with MTP Taq DNA Polymerase and 10X MTP Taq Buffer (Sigma, D7442-1500U) and the following cycling conditions: 94°C for 1min, followed by 30 cycles of 94°C for 1min, 65°C for 1min, and 72°C for 1min, and a final elongation step at 72°C for 10min. Illumina sequencing was performed in collaboration with the GeT core facility (Toulouse). Paired-end read assembly, quality and length filtering, OTU picking (97% sequence identity threshold) and chimera removal were performed with UPARSE [38]. OTUs with low counts (<0.1% of the total number of sequences per sample) were excluded. Sequences of samples with over 6000 reads were loaded into the QIIME 1.9.1 pipeline for diversity analysis [39]. Taxonomy assignment was performed with the SILVA database 132 (https://www.arb-silva.de/). Alpha diversity of bacterial communities was assessed from four different indexes including richness and/or evenness (Chao1, Shannon, Simpson and evenness). The Kruskal–Wallis test was used to estimate alpha diversity differences between groups. Beta diversity was used to analyse the dissimilarity between the groups’ membership and structure. Accordingly, abundance-weighted and/or phylogenetic-weighted distance matrices were generated on the basis of Bray-Curtis and weighted/unweighted UniFrac distances and reported according to principal coordinate analysis (PCoA). Permutational analysis of variance (PERMANOVA with 999 permutations) was used to determine significant differences between groups. Significance testing for taxon abundance was performed with a Wilcoxon rank sum test and the Bonferroni procedure to correct p-values. P-values ≤0.05 were considered significant.

### Faecal short-chain fatty acid (SCFA) concentration

Weighted faecal samples were reconstituted in 200μl Milli-Q water, disrupted, incubated at 4°C for 2h and centrifuged at 12000g at 4°C for 15min. Supernatants were weighed and saturated phosphotungstic acid solution was added (1g for 100μL). After overnight incubation at 4°C, samples were centrifuged again and SCFA concentrations were determined by gas chromatography (Nelson 1020, Perkin-Elmer, St Quentin en Yvelines, France) as previously described [40].

### Protein extraction and western blotting

Adipose tissue (subcutaneous and visceral) and colon samples were homogenized in 500μl of lysis buffer (Tris 25mM, EDTA 1mM, EGTA 5mM, MgCl2 0.1mM, Glycerol 10%, NaCl 150mM, Nonidet P-40 1%, SDS 1%) supplemented with freshly added protease inhibitor cocktail (cOmplete, Mini, EDTA-free Protease Inhibitor Cocktail, Roche), Sodium Orthovanadate (1mM), PMSF (1mM) and N-Ethylmaleimide (5mM). The homogenates were then centrifuged at 10 000 rpm at 4°C for 5min. A small aliquot (20μl) was used for protein concentration with the DC Protein Assay (Bio-Rad, USA). The rest was frozen at -80°C until use.

Proteins were separated on 12% SDS-PAGE gels, transferred to nitrocellulose membranes, and blocked with 5% BSA in Tris buffered saline (pH 8) containing 0.05% Tween 20 (TBST) at room temperature under agitation for 1h. Membranes were then incubated with diluted primary antibodies against phospho-HSL (Cell Signaling Technology), occludin (1:500 dilution; 33–1500; Invitrogen) or ZO-1 (1:500 dilution; 61–7300; Invitrogen) at 4°C under agitation overnight. After three washes with TBST, membranes were incubated with secondary antibodies in TBST at room temperature under agitation for 1h. Antibody interactions were detected with the Enhanced Chemiluminescence Detection Kit (Amersham Biosciences, RPN2108) followed by the Bio-Rad ChemiDoc imaging system. Data for pHSP were normalised to total protein loading using the Stain-Free Blot system (Bio-Rad, USA). ZO-1 and occludin protein contents were normalised to GAPDH expression. Band densities were analysed with Image J software.

### Quantitative real-time PCR

Total RNA was extracted from adipose tissues using TRIzol (Invitrogen, Life Technologies) and was reverse transcribed using the High Capacity cDNA Transcription Kit (Applied Biosystems, Life Technologies). Expression of the genes encoding α AR and β AR was analysed with the SYBR Green qPCR Master Mix (applied biosystems) and a CFX Bio-Rad system. The fold induction was calculated using the *Ct* method as follows: ΔΔ*Ct* = (*Ct*_target gene_- *Ct*_housekeeping gene_) _treatment_ - (*Ct*_target gene_- *Ct*_housekeeping gene_)_nontreatment_, and the final data were derived from 2^−ΔΔ*C*T^.

### Statistical analysis

All statistical analyses were performed with Statistica software (version 12). Data were presented as the mean ± standard deviation (SD). Normal data distribution was tested using the Kolmogorov–Smirnov test and the homogeneity of variance from the F-test. In the absence of normal distribution or variance homoscedasticity, the data were log-transformed before analysis. A one-way ANOVA (group effect) or ANOVA with repeated measures was used to determine significances (time (T) and group (G) effects & G x T interactions), followed by a Newman–Keuls post-hoc test when a significant effect was found. To assess beta- diversity, distance matrices between samples were generated on the basis of Bray-Curtis and weighted/unweighted UniFrac distances and reported according to principal coordinate analysis (PCoA). Analyses and graphical outputs were performed in R version 3.3.2. Differences with a p value ≤0.05 were considered statistically significant.

## Results

### HIIT is a time- efficient strategy to decrease total and epididymal fat mass

**Fig 1:**
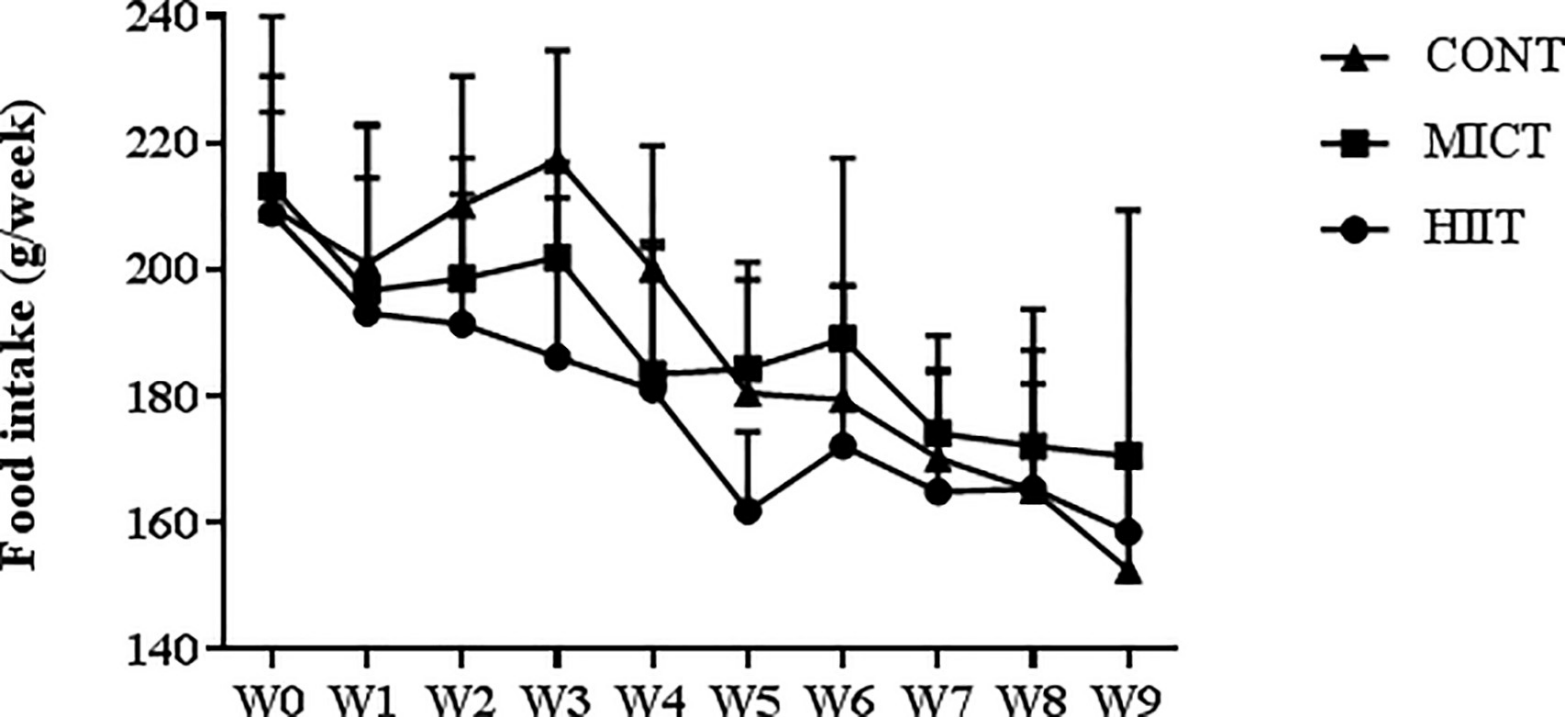
Weekly food intake. Data are expressed as the mean ± SD.

After 1 week of treadmill acclimatization, adult Zucker rats were randomly divided in three groups: HIIT (n = 12), MICT (n = 12) or CONT (no exercise) (n = 12). No significant difference in food intake between groups was observed during the study period (Fig 1).

**Fig 2:**
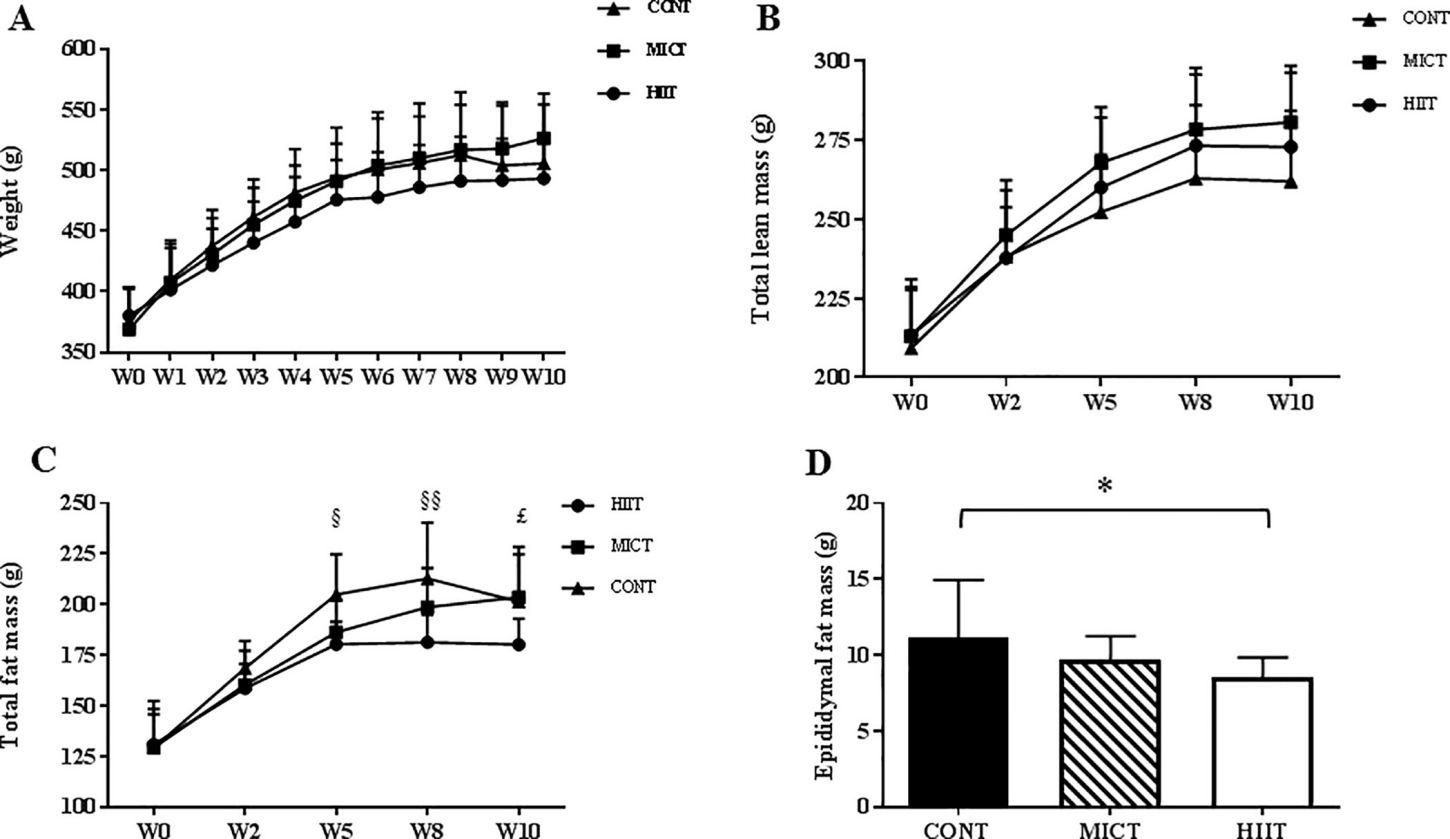
Effect of 10 weeks of exercise training on weight and body composition. Body weight (A), total lean mass (B), total fat mass (C) and epididymal fat mass (D). Data are expressed as the mean ± SD. § p ≤0.05 CONT vs. HIIT; § § p ≤0.01 CONT vs. HIIT £ p ≤0.05 HIIT vs. MICT and *p ≤0.05.

Despite our attempt to match running distances between groups, rats in the MICT group ran greater distances (23.3 ± 1.1 vs 21.4 ± 2.3 km) (p ≤0.05) and for a longer time (1941 ± 88 vs 1510 ± 114 min) (p ≤0.001) than rats in the HIIT group. After 10 weeks of training, body weight and total lean mass were comparable between groups (Fig 2A and 2B). However, total FM was lower in the HIIT than in the CONT group at week 5 (p ≤0.05) and week 8 (p ≤0.01), and in the HIIT group than in the MICT group at the end of the training protocol (p ≤0.05) (Fig 2C). At the end of the study, epididymal (visceral) adipose tissue was reduced only in the HIIT group (p ≤0.05) (Fig 2D).

### Gut microbiota composition and faecal short chain fatty acid concentration are not modified by exercise training

**Fig 3:**
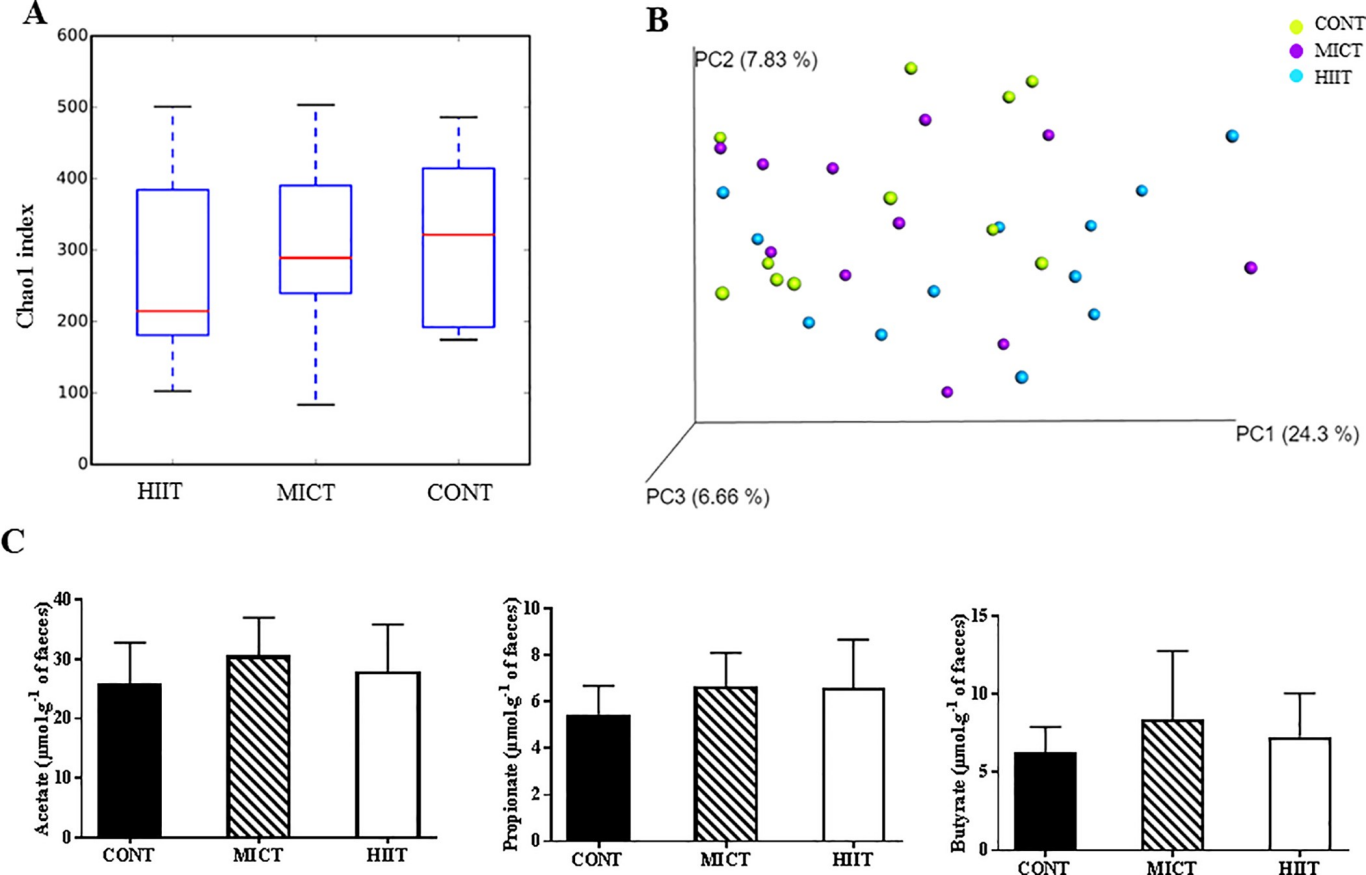
Effect of 10 weeks of exercise training on gut microbiota and faecal concentration of short-chain fatty acids (SCFAs). Gut microbiota composition: Chao1 index (A); Principal Component Analysis (PCoA) of the Bray-Curtis distances (β-diversity) (B); SCFA concentration in faeces after 10 weeks of training (C). Data are expressed as the mean ± SD.

To investigate the effect of the two exercise modalities on gut microbiota composition, the eubacterial 16S rRNA genes present in colon tissue were sequenced. All alpha diversity indexes (only Chao1 index is shown in Fig 3A) did not show any significant difference in species richness between the three groups at the end of the training programme (week 10). Beta-diversity analysis based on the uniFrac distance coupled with principal coordinate analysis (PCoA) showed no clustering of samples (Fig 3B). Univariate analysis of the abundance of major taxons (relative abundance >1%) %) identified no significant differences between groups. Similarly, the faecal concentrations of the major SCFAs (butyrate, acetate and propionate) were not significantly different in the three groups (Fig 3C).

### HIIT stimulates tight junction protein synthesis more efficiently than MICT, but both training modalities decrease plasma lipopolysaccharide binding protein (LBP)

**Fig 4:**
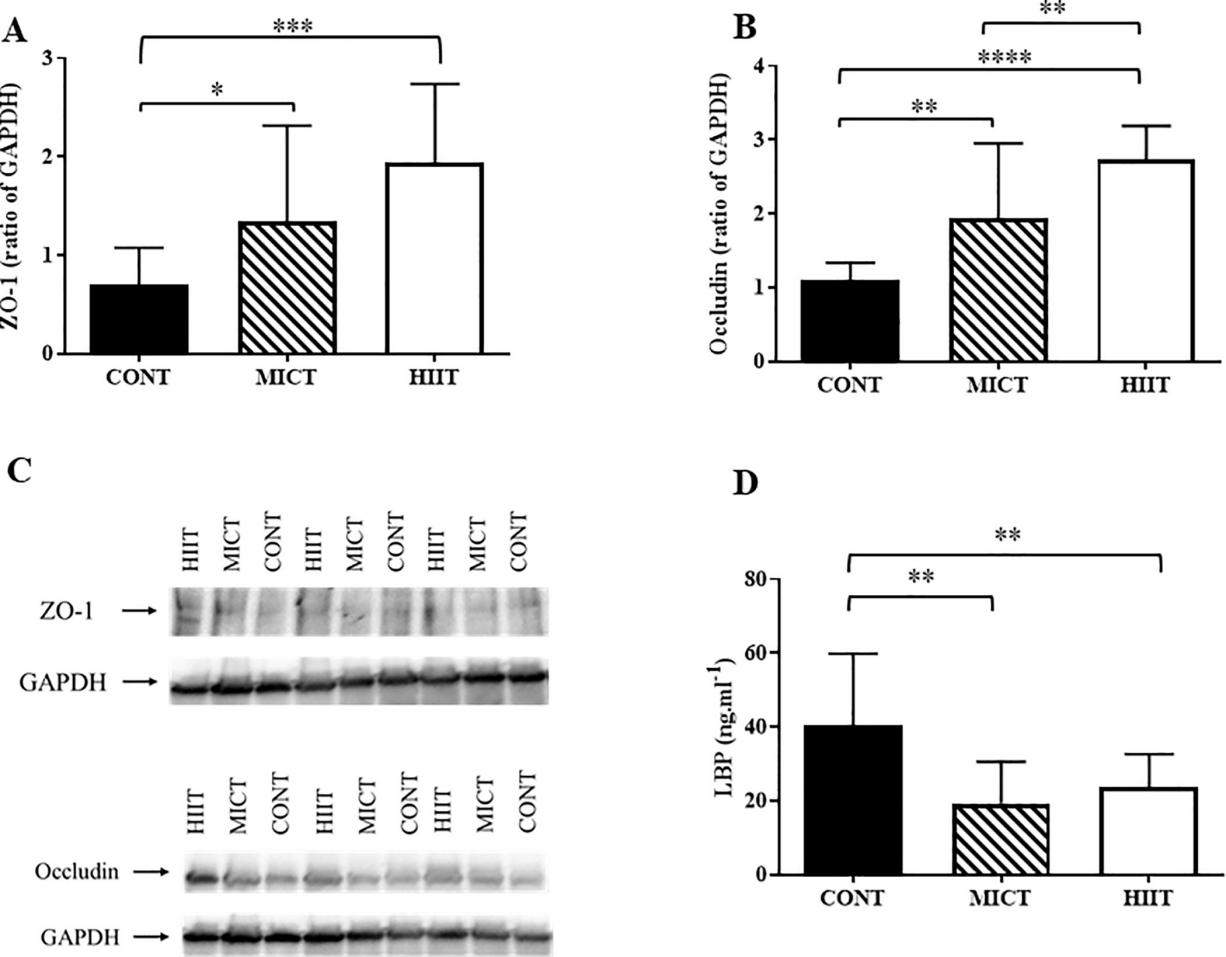
Effect of 10 weeks of training on intestinal permeability. Zonula occludens-1 (ZO-1) (A) and occludin (B) expression were assessed by western blotting. Representative images of the western blot results (C). Plasma levels of lipopolysaccharide binding protein (LBP) determined by ELISA (D). Data are expressed as the mean ± SD; *p ≤0.05, **p ≤0.01 and ***p ≤0.001.

At the end of the training programme, zonula occludens-1 (ZO-1) (MICT vs CONT; p ≤0.05 and HIIT vs CONT; p ≤0.005) and occludin (MICT vs CONT; p≤ 0.01 and HIIT vs CONT; p ≤0.001) were upregulated in both training groups, particularly in the HIIT group (Fig 4A, 4B and 4C). In addition, occludin expression level was significantly correlated with epididymal adipose tissue depots (r = -0.5; p ≤0.05) and total FM change (FM at week 10—FM at baseline) (r = -0.4; p ≤0.05). Occludin and ZO-1 were also negatively related to IL-6 secretion in epididymal adipose tissue (r = -0.4, p ≤0.05 for both). Finally, expression of LBP (a marker of obesity [41,42] and metabolic endotoxemia [43]) was reduced by about 50% in both HIIT and MICT groups (p≤ 0.01 vs CONT) (Fig 4D).

### MICT and HIIT induce anti-inflammatory effects at the systemic and adipose tissue levels

**Fig 5:**
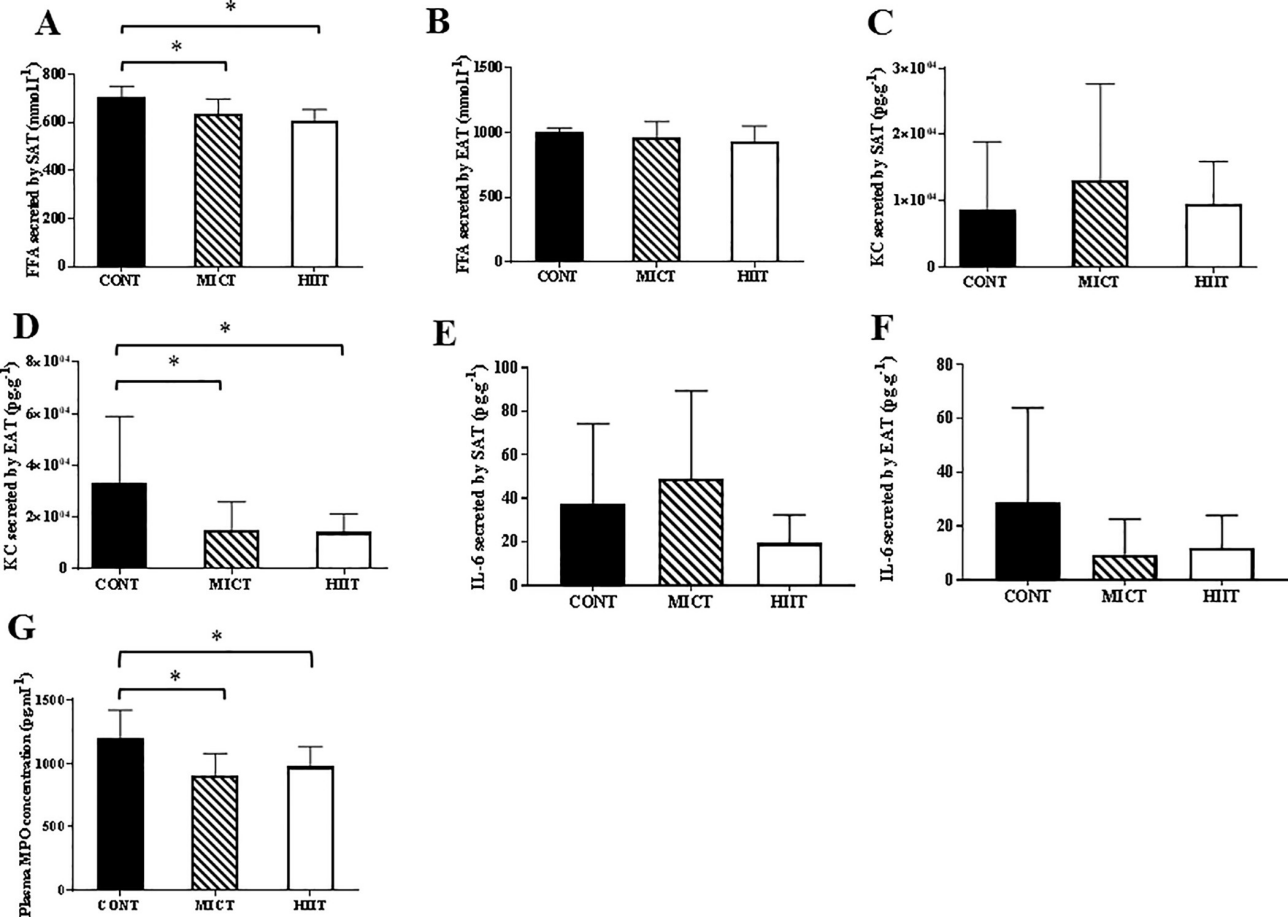
Effect of 10 weeks of exercise training on adipose tissue and systemic inflammation. Free fatty acid (FFA) secretion by subcutaneous adipose tissue (A) and visceral adipose tissue (B). Keratinocyte-derived chemokine (KC) secretion by subcutaneous adipose tissue (C) and visceral adipose tissue (D). IL-6 secretion by subcutaneous adipose tissue (E) and visceral adipose tissue (F). Plasma myeloperoxidase (MPO) concentration (G). Data are expressed as the mean ± SD. *p ≤0.05 vs CONT.

At the end of the training programme (week 10), secretion of free fatty acids (FFA) was reduced in subcutaneous adipocytes (p ≤0.05 HIIT and MICT vs CONT), but not in the epididymal tissue (Fig 5A and 5B). Conversely, keratinocyte-derived chemokine (KC) secretion was reduced in the epididymal adipose tissue (p ≤0.05 HIIT and MICT vs CONT), but not in subcutaneous adipocytes (Fig 5C and 5D). On the other hand, IL-6 secretion in the two types of adipose tissue was not significantly different from that in the CONT group (Fig 5E and 5F). At the systemic level, the plasma concentration of myeloperoxidase (MPO) decreased in both MICT and HIIT groups after 10 weeks of training (p ≤0.05 vs CONT) (Fig 5G). Plasma MPO levels were positively associated with plasma LBP levels (r = -0.5; p ≤0.001).

### Only HIIT increases the α2 AR/β3 AR ratio in subcutaneous adipose tissue

**Fig 6:**
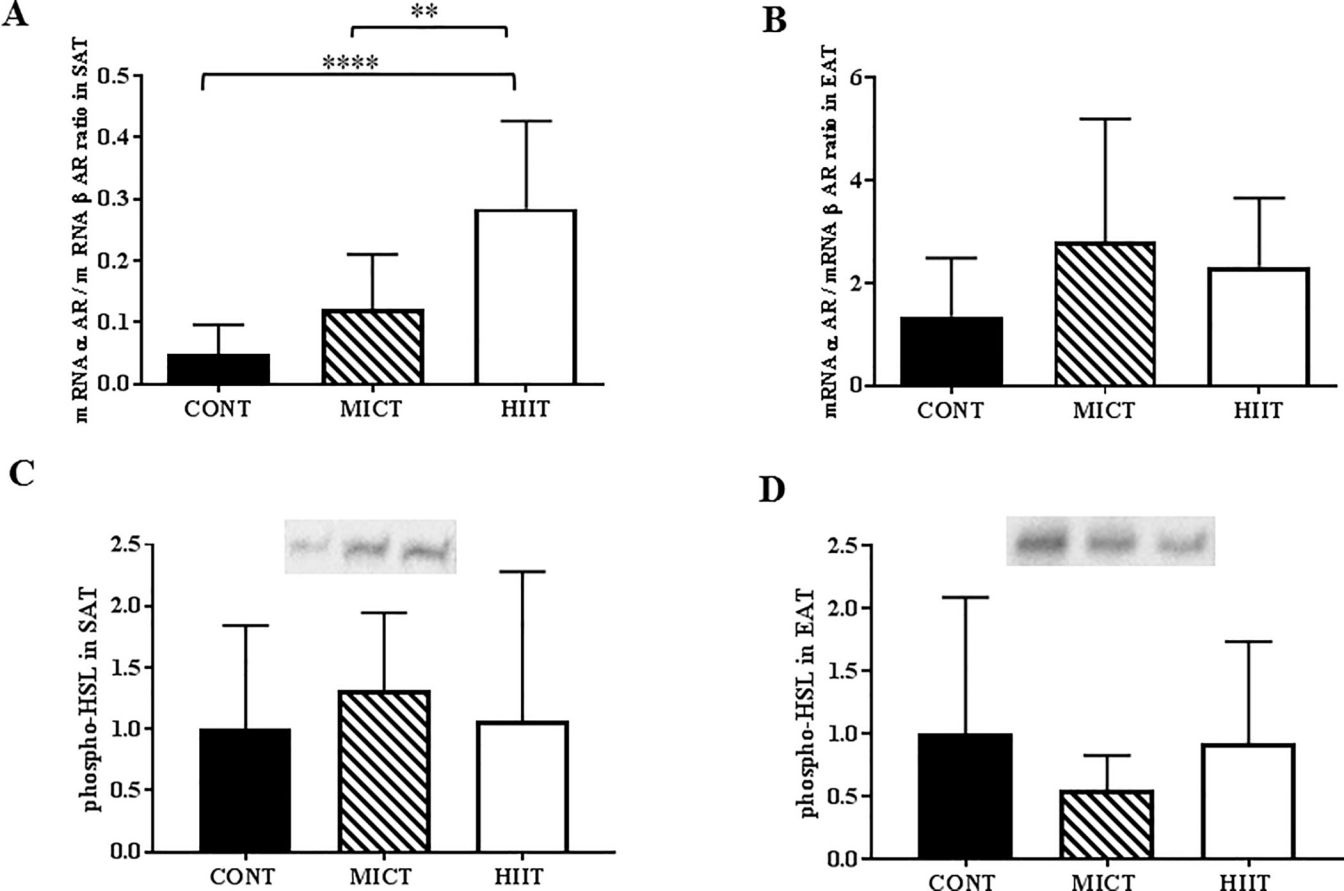
Effect of 10 weeks of exercise training on lipolysis factors. The α-adrenergic receptor/β adrenergic receptor (α AR/β AR) ratio determined by q-PCR in subcutaneous adipose tissue (A) and epididymal adipose tissue (B). Level of phosphorylated hormone-sensitive lipase (phospho-HSL) in subcutaneous adipose tissue (C) and epididymal adipose tissue (D). Data are expressed as the mean ± SD; **p ≤0.01 and ***p ≤0.001.

At the end of the training programme, the α/β adrenergic receptor RNA ratio in subcutaneous adipose tissue was greater in the HIIT group than in the CONT (p ≤0.001) and MICT (p ≤0.01) groups, but not in epididymal adipose tissue (p ≥0.05) (Fig 6A and 6B). The level of phosphorylated hormone-sensitive lipase (phospho-HSL) in subcutaneous (Fig 6C) and epididymal adipose tissue (Fig 6D) was not modified by exercise training.

### HIIT and MICT improve glucose metabolism, but do not modify the lipid profile

**Fig 7:**
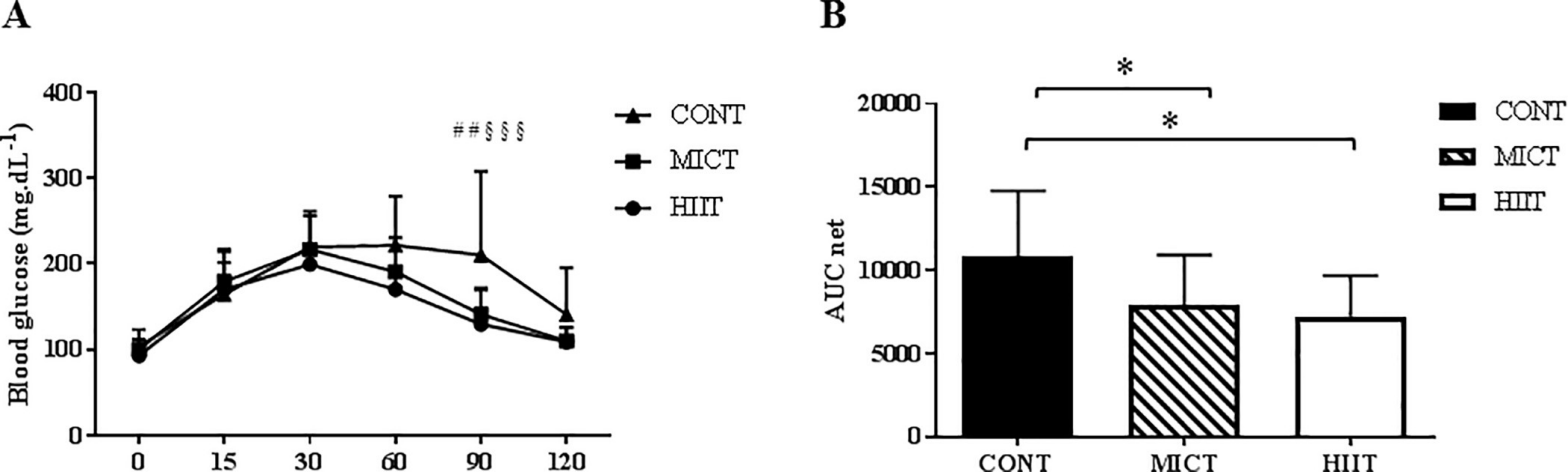
Effect of 10 weeks of exercise training on glucose tolerance. Blood glucose monitoring during the oral glucose tolerance test (A) and net area under the curve (_net_AUC) for glucose (B) at the end of the 10 weeks of exercise training in the three groups. Data are expressed as the mean ± SD. ## p ≤0.01 CONT vs MICT; § § § p ≤0.001 CONT vs HIIT and *p ≤0.05.

Fasting glycaemia did not differ between the three groups at the end of the study (week 10) (Table 1). Repeated ANOVA measures of the results of the oral glucose tolerance test (OGTT) performed at the end of the training programme showed a significant time x group interaction at 90min (p ≤0.01) with higher values in the CONT than in the MICT and HIIT groups (Fig 7A). The net area under the curve (_net_AUC) was lower in the MICT and HIIT groups than in the CONT group (p ≤0.05) (Fig 7B). After 10 weeks, the plasma level of LDL cholesterol was higher in the MICT than in the CONT group (p ≤0.05). Conversely, plasma total cholesterol, HDL cholesterol, triglycerides and FFA were not modified by physical training (Table 1).

**Table 1:** Lipid and glycaemic profiles after 10 weeks of training (mean ± SD).

	Total Chol (mg.dL^-1^)	LDL (mg.dL^-1^)	HDL (mg.dL^-1^)	TG (mg.dL^-1^)	FFA (mmol.L^-1^)	Insulin (ng.mL^-1^)	Glucose (mmol.L^-1^)	HOMA-IR
**CONT**	227.1 ± 49.9	260.2 ± 74.0	69.0 ± 36.0	603.0 ± 470.6	556.5 ± 442.4	2.5 ± 1.6	5.7 ± 1.1	17.4 ± 10
**MICT**	217.8 ± 33.4	314.5 ± 43.7^#^^£^	80.2 ± 19.3	426.5 ± 155.8	415 ± 141.7	3.4 ± 3.6	5.6 ± 0.6	25.0 ± 31.1
**HIIT**	209.2 ± 40.2	270.1 ± 52.5	74.9 ± 26.6	450.8 ± 94.9	493.9 ± 163.9	2.6 ± 3.7	5.3 ± 0.7	17.1 ± 23.1

Chol, cholesterol; LDL, low-density lipoproteins; HDL, high-density lipoproteins; TG, triglycerides; FFA, free fatty acids; HOMA-IR, homeostatic model assessment of insulin resistance.

# p ≤0.05 CONT vs MICT

£ p ≤0.05 MICT vs HIIT

### HIIT and MICT modify the resting respiratory exchange ratio, but not the mean daily spontaneous physical activity and energy intake

After 10 weeks of training, the mean daily total energy expenditure and food intake (for the 12h-light period and during 24h) were comparable in the three groups (Table 2). Conversely, for the 12h-dark period, spontaneous physical activity was increased in the two training groups (p ≤0.05), and energy intake was higher in the MICT than in the CONT group (p ≤0.05).

At the end of the study, the respiratory exchange ratio (RER), evaluated by indirect calorimetry in mice housed in calorimetry cages for 24h, was significantly higher in the two training groups (0.97 ± 0.02 in the HIIT and 0.97 ± 0.02 in the MICT vs 0.93 ± 0.05 in the CONT group; p ≤ 0.05) (Table 2).

**Table 2:** Indirect calorimetry results after 10 weeks of training (mean ± SD).

	24 hours				12h-light (08:05–20:00)				12h-dark (20:05–8:00)			
	EE/LM (kJ/g)	RER	Spontaneous activity (m)	Food intake (g)	EE/LM (kJ/g)	RER	Spontaneous activity (m)	Food intake (g)	EE/LM (kJ/g)	RER	Spontaneous activity (m)	Food intake (g)
HIIT	0.93 ± 0.05	0.97 ± 0.02	211.1 ± 40.9	22.31 ± 3.3	0.50 ± 0.03	0.97 ± 0.03	131.1 ± 31.0	13.69 ± 2.2	0.42 ± 0.02	0.96 ± 0.03	73.9 ± 12.9	7.91 ± 1.8
MICT	0.93 ± 0.08	0.97 ± 0.02	184.9 ± 49.0	21.79 ± 2.7	0.50 ± 0.05	0.97 ± 0.03	112.4 ± 28.8	13.78 ± 2.7	0.43 ± 0.03	0.96 ± 0.02	67.6 ± 21.9	8.36 ± 1.41
CONT	0.95 ± 0.12	0.93 ± 0.05^#^^$^	171.8 ± 46.5	19.05 ± 4.2	0.51 ± 0.07	0.94 ± 0.04	113.5 ± 37.9	12.57 ± 2.9	0.43 ± 0.06	0.91 ± 0.06^#^^$^	50.5 ± 14.8^#^^$^	6.36 ± 2.5 ^#^

EE: energy expenditure, LM: Lean mass, RER: respiratory exchange ratio.

**#** p ≤0.05 CONT *vs*. MICT

$ p ≤0.05 CONT *vs*. MICT; p ≤0.05 MICT *vs*. HIIT

## Discussion

This study in Zucker rats, a genetic model of obesity, confirms that HIIT is more efficient than MICT in decreasing total FM, in particular visceral adipose tissue, which is responsible for numerous metabolic complications. In contrast, both exercise training modalities reduced systemic and adipocyte inflammation and improved glucose metabolism. Finally, endotoxemia reduction was comparable in both training groups (LPB expression), although HIIT increased the synthesis of tight junction proteins to a greater extent than did MICT. These positive adaptations observed in the HIIT group, in which rats ran shorter distances than those in the MICT group, demonstrate the time efficiency of this exercise modality.

MICT protocols are still traditionally recommended for sedentary overweight or obese individuals to reduce FM. However, a growing body of evidence shows that HIIT can be a more amusing and time-efficient exercise modality to lose total and visceral adipose tissue [21,22,44]. Our present results in obese Zucker rats confirm that HIIT (6 sets at 18 m.min^-1^ for 4min followed by 3min at 10 m.min^-1^ for 10 weeks) significantly reduces total and epididymal FM compared with MICT (12 m.min^-1^ for 51min for 10 weeks). Other studies also demonstrated a greater effect of HIIT on total and abdominal FM loss than with MICT in different animal models of obesity. Wang et *al*. showed that in mice fed a high-fat diet, the adiposity index (44% and 53%, respectively) was lower in the HIIT (10 x 4min [85–90% VO_2_max]/2min active rest [5 m.min^-1^]) than in the MICT group (65–70% of VO_2_max [distance-matched continuous running]) [45]. Similarly, in male Sprague-Dawley rats fed a high-fat diet, HIIT (30sec [32 m.min^-1^ ]/10sec passive recovery, 5° slope) decreased epididymal FM, whereas MICT (16 m.min^-1^ for 40min, 5° slope) had no effect [46]. In the sole study, to our knowledge, to assess the effects of an HIIT protocol (4min at 65–80% of VO_2_ max/3min at 50–65% of VO_2_ max) in Zucker rats, the authors observed reduced FM and abdominal fat pad in the HIIT group (5.7±0.2 *vs*. 6.4±0.2g in the control group without exercise) [35].

However, the mechanisms by which HIIT decreases abdominal and in particular visceral FM are still unknown. Here, we assessed the potential role of the gut microbiota in such adaptations. In our study, exercise (MICT and HIIT) did not have any effect on gut microbiota composition (quantity and function). In addition, the main faecal SCFAs (acetate, propionate and butyrate) were not modified by the two exercise modalities. This result is surprising because previous studies have often reported that bacterial richness and function are improved by regular physical activity, and that some bacterial species related to the anti-inflammatory profile, health and leanness could also be increased by exercise [27,28,30,31,47–49]. To investigate the effect of exercise on gut microbiota, MICT training or spontaneous exercise (spontaneous activity wheel) are generally used, and only two studies have tested the effect of a HIIT programme on gut microbiota modulation. The first one found an increase in the Bacteroidetes/Firmicutes ratio and in *Lactobacillus*, *Bacteroidales* and *Dorea* species in mice after HIIT training, without any change in the epididymal FM, suggesting there is no correlation between these variables [33]. The second study reported slight differences in gut microbiota composition in rats undergoing MICT and HIIT (increase in *Parasutterella excrementihominis* and *Lactobacillus johsonii* in the MICT group, and in *Clostridium saccharolyticum* in the HIIT group). Unfortunately, the authors did not measure total and visceral FM [32]. In Zucker rats, only one study has assessed modulation of gut microbiota after an MICT training programme (12.5 m.min^−1^ for 30min, 5 days per week for 4 weeks). At the end of the training period, the authors found that the composition of the gut microbiota had changed [26]. However, this analysis concerned only faecal samples of three animals/group, whereas in our study, 16S sRNA gene expression in colon tissue was investigated, which is a more suitable assay [50,51].

In our study, the extreme amount of adipose tissue in Zucker rats could mask the changes in gut microbiota composition induced by regular exercise [23,52,53]. Nevertheless, Lamoureux et *al*. observed minor effects of spontaneous exercise on gut microbiota composition also in normal-weight C57BL/6 mice [24]. Hence, other factors than weight or total FM could explain the absence of modulation of gut microbiota after MICT or HIIT in our study. As most of the publications on exercise and gut microbiota are observational studies, the potential links between physical activity and gut microbiota modulation are still unknown and mechanistic investigations in animal models are needed. The lack of protocol standardization for gut microbiota analysis (DNA extraction, regions analysed, bioinformatics analysis *etc*.) could also complicate interpretation of the results. In addition, following a perturbation, gut microbiota can return to its initial functional or taxonomical composition following a perturbation, which is defined as resilience. As we did not analyse gut microbiota during the training programme, we do not know whether gut microbiota is resilient or resistant to training interventions.

Intestinal permeability is regulated by the tight-junction proteins claudin, occludin, and ZO-1. In obesity, their expression, localization and distribution are altered [7], leading to an association between obesity (FM) and intestinal permeability [54]. In our study, the colon expression of occludin and ZO-1 was increased at the end of the HIIT and, to a lesser extent, the MICT programme. Moreover, the amount of epididymal adipose tissue was negatively associated with their expression level, reinforcing the beneficial effect of HIIT. Holland et *al*. showed that 10 days of exercise in Sprague–Dawley rats (30m.min^-1^ for 60min, 5 days/week) reduces 24h post-exercise intestinal inflammation and reinforces the intestinal barrier function [55]. In agreement, our study showed a negative correlation between the expression of occludin and ZO-1 and IL-6 secretion by the epididymal adipose tissue. Moreover, secretion of KC (the analogue of human IL-8) was decreased in the epididymal adipose tissue. In humans, plasma IL-8 levels are high in obese individuals and are associated with abdominal adiposity and insulin sensitivity [56]. Neels et *al*. showed in genetically and diet-induced obese mice an increase in KC in the blood and in the epididymal adipose tissue [57]. They also found that after KC treatment, adipogenesis is not directly affected, but inflammatory factors (MCP-1, IL-6, TNF-α) are increased in adipose tissue [57], leading to low-grade systemic inflammation. Similarly, we observed that HIIT and MICT can decrease systemic inflammation, as indicated by the lower levels of MPO, a biomarker of inflammation and cardiovascular risks [58].

Intestinal permeability promotes metabolic endotoxemia, which is defined as an increase in LPS plasma levels [16,59]. When LPS is in the bloodstream, it is recognized by LBP and forms LBP-LPS complexes. LBP levels are correlated with abdominal FM[60], making of LBP a good obesity marker [41,42]. In our study, plasma LBP was similarly reduced by MICT and HIIT. This positive exercise effect is supported by the finding that plasma LPS is reduced in male Wistar rats after chronic (1h/day, 5 days/week, for 8 weeks) and acute swimming exercise (two 3h bouts, separated by a 45min rest period) [61].

A second hypothesis concerning the greater impact of HIIT on visceral fat mass loss could be related to a greater lipolytic activity. Adipocyte lipolysis is regulated by pro-lipolytic pathways mediated by the β AR and natriuretic peptide receptors and by anti-lipolytic pathways via α AR and insulin through insulin receptor substrate-1 (IRS-1). As β AR expression is higher in visceral than in subcutaneous adipose tissue [62], the higher catecholamine production induced by HIIT [63] could explain the greater reliance on visceral FM during HIIT. In our study, HIIT (but not MICT) increased the α AR/β AR mRNA ratio in subcutaneous adipose tissue, suggesting a greater anti-lipolytic activity. Although we expected a greater lipolytic effect in the visceral than in subcutaneous adipose tissue, this result is interesting. Indeed, a greater ability to increase subcutaneous adipose tissue can prevent FM ectopic deposition and visceral adipose tissue development, as previously described. Moreover, although the α AR/β AR mRNA ratio in visceral adipose tissue was not changed by physical training, the receptor sensitivity could be modified. Exercise concomitantly reduces α AR sensitivity and increase β AR sensitivity, as already shown in the subcutaneous adipose tissue [64,65] of obese subjects. To evidence a possible increase in lipolytic activity in visceral adipose tissue, we measured the phosphorylation level of HSL. HSL and adipose triglyceride lipase (ATGL) are responsible for more than 90% of triglyceride hydrolysis [66], but only HSL induces PKA-dependent lipolysis [67]. However, the amount of phospho-HSL was not influenced by physical activity.

Both exercise modalities (MICT and HIIT) decreased the glucose AUC_net_ after OGTT and improved glucose utilization at rest (as shown by the respiratory exchange ratio at the end of the study). The improved glucose metabolism in our study can be partly explained by the reduction of KC secretion in the adipose tissue and by the decrease in LBP levels [57,61]. The positive association between LBP and the glucose AUC_net_ supports this hypothesis (r = 0.4).

The present study has certain limitations that should be considered. First, MICT and HIIT protocols were not based on the animals’ VO_2_ consumption and VCO_2_ production. The protocols were originally designed to have the same total running distance for all groups as implemented by Metz et al. in 2005 [34]. The intensity and the duration chosen for each modality had already been adopted in other studies [34–36]. Second, diet composition did not strictly comply with the feeding recommended by the American Institute of Nutrition (AIN-93M) [68]. The three groups nevertheless received the same diet preparation (SAFE 04), and the differences in body composition and metabolic profiles observed between groups were therefore induced by physical activity. A third possible limitation concerns the time spent (48h) in metabolic chambers to determine energy expenditure and substrate oxidation. Owing to the number of animals, it was not feasible for us to keep the rats for a period of 72h as usually recommended. However, we took care to start the measurements after 24h of acclimatization, and all the groups were compared in the same conditions. Fourth, considering the model of obesity used (Zucker rat), it would have been interesting to determine the plasma level of leptin. However, the total amount of blood recovered after sacrifice required us to make certain choices. A lack of leptin response has been documented in obese Zucker rats [69], which carry a mutation (fa) in the leptin receptor gene [70]. Thus, even though leptin is produced, its effect is blunted by the mutation. The last limitation of our study concerns the statistical methods used with such a small sample of animals. In the absence of ‘normal distribution’ and ‘variance homoscedasticity’, we elected to transform the data with log-transformation and not by nonparametric tests since repeated values were compared. After transformation, the Newman-Keuls post hoc test was maintained for analysis.

In conclusion, in this genetic model of obesity, HIIT led to a reduction in total and visceral FM more efficiently than MICT. Contrary to our initial hypothesis, gut microbiota composition is not involved in exercise-induced FM loss in obese Zucker rats. The decrease in FM could be explained by specific changes in the lipolysis pathway, including the sensitivity of adrenergic or insulin receptors, lipase activities or mitochondrial adaptations. On the other hand, the excess of adipose tissue in Zucker rats could limit gut microbiota modulation, suggesting that diet-induced obesity models would be more suitable to investigate the potential link between FM loss and gut microbiota. Finally, despite the lack of HIIT-induced changes in microbiota composition, we found that both training methods (MICT and HIIT) promoted total FM loss and decreased inflammation, improving intestinal permeability, decreasing LBP and enhancing glucose metabolism. As the HIIT programme was based on running a shorter distance for a shorter time, this modality is as a time-efficient strategy against obesity.

## Supporting information

S1 Table Data availability. (XLSX)
